# Parental involvement in paediatric cancer treatment decisions

**DOI:** 10.1111/j.1365-2354.2009.01116.x

**Published:** 2010-09

**Authors:** K McKenna, J Collier, M Hewitt, H Blake

**Affiliations:** 1 Department of Psychology, Nationwide Children's HospitalColumbus, OH, USA; 2 School of Nursing, Faculty of Medicine and Health Sciences, Queen's Medical CentreNottingham, UK; 3 Department of Child Health, Queen's Medical Centre Campus, University HospitalNottingham

**Keywords:** parental satisfaction, decision making, communication, paediatric oncology

## Abstract

This study investigated parents' information needs and involvement in decision-making processes affecting the care of children diagnosed with cancer. Interviews and questionnaires were used to assess parental satisfaction in 50 mothers and 16 fathers responsible for 58 children in an English Paediatric Oncology Unit. Parents reported that doctors contributed almost twice as much to the decision-making process as they did, but parental satisfaction was positively correlated with the amount of information provided when giving informed consent. Satisfaction about their involvement in this process relied heavily upon the level of support received from others. Parents consenting to their child's involvement in non-randomised trials perceived themselves to be under greater pressure from others during the decision-making process while those whose children were further along the treatment trajectory were more uncertain about decisions previously made. Findings indicate that the accessibility, support, information and degree of control afforded to parents by healthcare professionals impacts upon their satisfaction with both the decision-making process and their confidence in the decisions thus made. Information and support tailored to parents' specific needs may therefore enhance satisfaction with clinical decision making and reassure parents about decisions made in the long-term interest of their child's health.

## INTRODUCTION

Rapidly evolving developments in medical technology have provided more options for the treatment of different malignancies. As new medications are tested for their effectiveness, more paediatric patients are recruited into treatment programmes such as clinical trials (Smith *et al.* 1993; Van Stuijvenberg *et al.* 1998). Consequently, the informed consent process has become a major issue in the field of paediatric oncology and the emphasis is shifting towards ‘shared consent’ to provide parents with the appropriate levels of information and support to assist their participation in decision making (Van Stuijvenberg *et al.* 1998; Massimo *et al.* 2004). To this end, partnership research with parents of paediatric patients has been conducted to improve the informed consent procedure (Eder *et al.* 2007) and strategies are being developed to facilitate the parental decision-making process (Tomlinson *et al.* 2006). Although research in this area is rapidly increasing, studies that seek to determine why parents of children opt to participate in intensive treatments regimens (including randomised clinical trials) and the residual effects of these decisions have been limited. Current research shows a range of factors associated with parental involvement in decision making including their relationship with the physician, the nature of physician–parent communication, trust, parents' and physician's knowledge and experience, and the perceived importance of the parental role (Pyke-Grimm *et al.* 2006).

Early studies have shown that even though parents may be generally satisfied with the handling of informed consent by medical practitioners, parental decisions about treatment are often made under high levels of stress (Ruccione *et al.* 1991). When interviewed several months after making their decision, parents report that they had been faced with an extensive amount of information during a time of considerable anxiety with a resultant feeling of a lack of control over the situation (Levi *et al.* 2000). Furthermore, parents report varying levels of choice and participation in clinical decision making (Levi *et al.* 2000), which suggests the need for a thorough evaluation of the way in which clinicians recruit participants, the information which is provided by medical staff to both the patient and their parents, and healthcare providers adherence to the principles of informed consent.

Determining which information to give to parents as well as the level of involvement they want in their child's treatment decisions can be a daunting task for paediatric oncologists (Nelson & Nelson 1992). Whitney *et al.* (2006) present a decisional model which suggests that clinicians may assume decisional priority when there is a single best medical choice, but they should encourage parents (and children when appropriate) to assume decisional priority when there are two or more clinically reasonable choices. Although an early study conducted by Pyke-Grimm *et al.* (1999) was one of the first attempts to ask parents the type of decision-making cooperation they would prefer, research is lacking regarding the perceived levels of involvement different parties have in the selection of a paediatric cancer treatment regimen. Similarly, parents' long-term satisfaction with their treatment decisions or involvement in the decision-making process is under-researched, giving consultants little insight into how they can identify the needs of both the patient and their parents when discussing various courses of treatment.

The current study aimed to identify parental perceptions of both their own involvement and that of others in the decision to enrol their child on to a specific cancer treatment protocol and their satisfaction with this decision. The hypotheses were as follows:

1. If parents do not have the level of involvement they prefer in deciding on the enrolment of their child in a treatment protocol, they will have lower satisfaction in the short and long term.
2. If parents receive too much or too little information from the medical staff during the decision-making process, they will have lower satisfaction in the short and long term.

## METHODS

### Procedures and measures

This study was approved by the local Ethics Committee. Parents were recruited from the Paediatric Oncology Unit of the Queen's Medical Centre in Nottingham, UK. Oncologists attained permission from the parents during or between clinic appointments and ward rounds. The purpose of the study in the context of examining parental satisfaction with the treatment decision-making process was explained to the parents by the oncologist and the research assistant before written consent was obtained. The research assistant met with the parents only after the medical staff obtained their initial verbal consent. The questionnaires were coded with a respondent number to protect the identity of the participants, thus ensuring their confidentiality.

Parents completed an abbreviated form of the Decisional Conflict Scale, which is based on decisional conflict in the medical setting regarding cancer screening and vaccination uptake (O'Connor 1995). The scale assesses three main factors including: (1) healthcare consumers' uncertainty in making a health-related decision; (2) the factors contributing to the uncertainty; and (3) healthcare consumers' perceived effectiveness in decision making. The scale is set at the equivalent of an eighth grade (13–14 years of age) reading level. This is based on a readability score, the ‘Flesch-Kincaid Grade Level’ score, which rates text on a US school grade level. Responses are marked on a Likert scale ranging from 1 (strongly agree) to 5 (strongly disagree).

The main themes of the scale and the eight questions subsequently chosen from it for this study, deal with uncertainty, vacillation, involvement of others, satisfaction and anticipated regret in the decision-making process. The test–retest reliability of the Decisional Conflict Scale in its full form (16 questions) is 0.81, with an internal consistency rating of 0.78–0.92.

**Figure 1:**
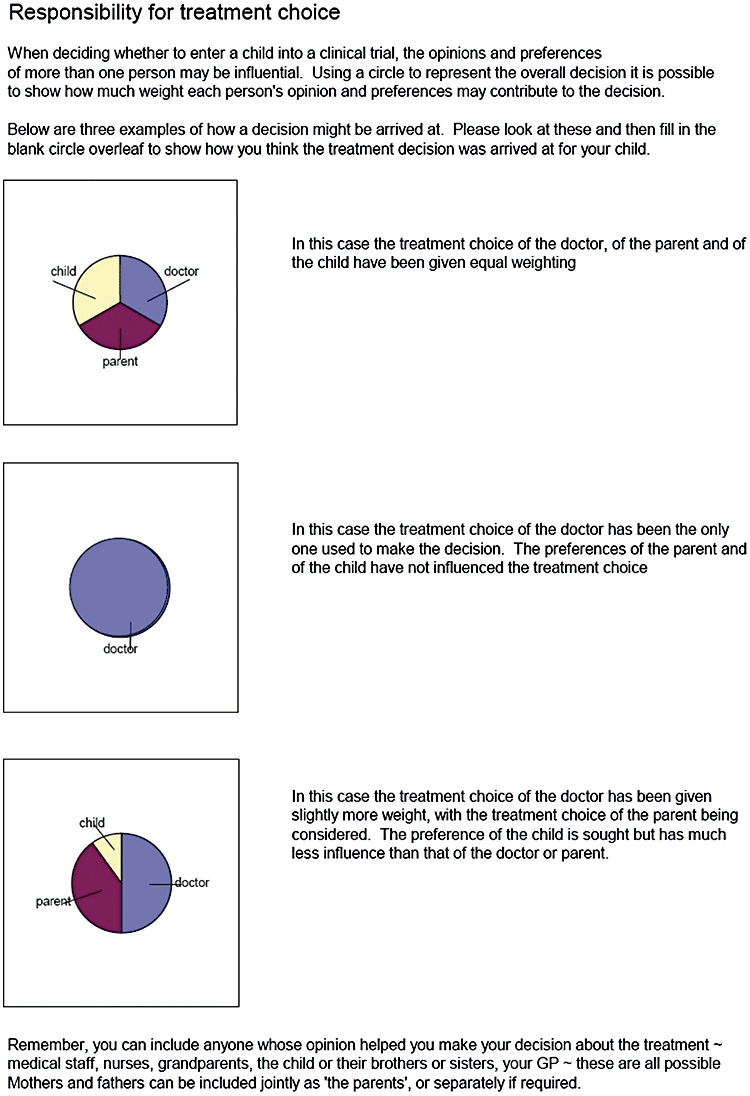
The Responsibility for Treatment Choice Questionnaire.

**Figure 2:**
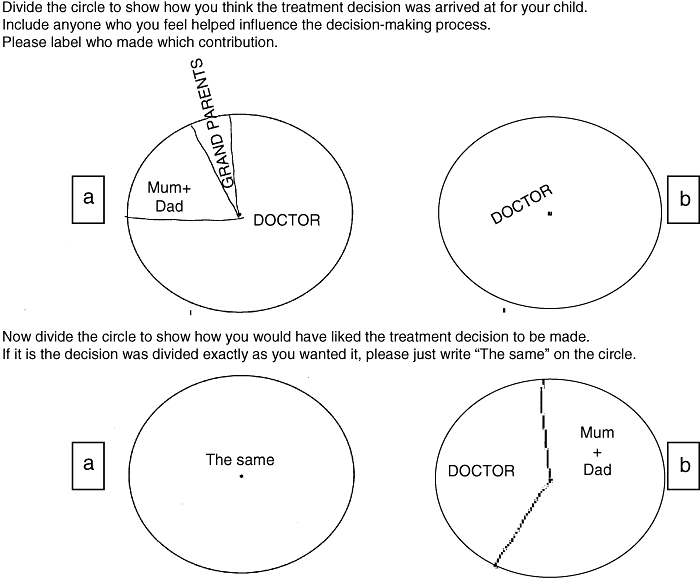
Completed forms for family (a) and family (b).

The parents also completed the ‘Responsibility for Treatment Choice’ Questionnaire whereby they divided a circle based on how they felt certain parties contributed to the treatment decision. They were instructed to identify as many people as necessary and divide the circle as many times that they felt appropriate. A second circle was divided based on how they would have preferred responsibility for the treatment decision to be distributed. If the treatment decision process occurred exactly as the parents desired, they wrote ‘The same’ on the circle. This scale has been used previously at the recruiting hospital to assess parent satisfaction in treatment decisions (see Figs 1 and 2).

The Revised Decisional Conflict scale has reverse scoring, such that higher scores indicate a higher degree of conflict. For the Responsibility for Treatment Choice Questionnaire, the percentage (A) of each division was calculated by the following equation: Adivision = (_/360) × 100. Data for the short interview were numerically coded (e.g. parental satisfaction with their involvement in the treatment decision process was coded in general levels, ranging from very dissatisfied (1) to very satisfied (5).

Previous knowledge and information preferences were also addressed via four additional questions which were administered to each parent by the research assistant to determine:

- whether they had heard of paediatric cancer treatments or research before, and if so, where;
- how happy they were with their involvement in their child's treatment decision (on a scale of 1–5);
- what additional information about treatment options they would have liked;
- how much time (in days) they had to make their decision.

The answers were noted at the time of the interview. Demographic data (such as the parents' age, occupation and education level) were collected by means of a short demographic questionnaire.

### Data analysis

Data were analysed using the Statistical Package for the Social Sciences, Version 10.1. The total sample of 66 parents was used in the analysis of the Decisional Conflict Scale, short interview questions and parental demographic data. To control for the potential weighting of scores from families in which both parents completed the Responsibility for Treatment Choice Questionnaire, only the mother's data were used from the pair for analysis. This resulted in a sample size of 58 parent–child dyads.

## RESULTS

Each of the 66 parents approached consented to take part in the study and there was no attrition from the study.

### Participant demographics

These are presented in Table 1. While all parents of 58 children were invited to take part, mothers were more likely than fathers to consent and complete the questionnaires. Parents were between 20 and 50 years of age (mean = 34.18). Their children ranged in age from <1 year to 16.5 years at diagnosis. Thirty parents had children who were diagnosed within the prior 12 months. The remaining 36 parents had children who were at least 1 year into their treatment protocol (i.e. 1 year post treatment decision). The range in time since diagnosis for the entire sample was 6 weeks to 11 years. Of the 58 children in the study, 30 (51.7%) were still undergoing treatment. The most common diagnoses were acute lymphoblastic leukaemia (*n*= 29; 50%), Wilms' tumour (*n*= 6; 10.1%), acute myelogenous leukaemia (*n*= 3; 5.2%) and non-Hodgkin's lymphoma (*n*= 3; 5.2%). Fifty-five parents (83.33%) had been asked to join a treatment trial. Eleven parents (16.67%) were given treatment options to choose from at the time of diagnosis, but were not part of a research protocol. Overall, 49 of the children had been in randomised treatment programmes and 17 on non-randomised protocols.

**Table 1:** Demographic data

Variable	
Age in years (range, mean)	
Parent	20–50 (34.18)
Child	0.83–16.69 (6.04)
Education	
Left school at 16 years (no GCSE/CSE O levels)	11
GCSE/CSE O levels	42
A levels	3
Vocational qualification	5
Graduate/postgraduate professional qualification	5
Time since diagnosis (months) (*n*, %)	
<12	30 (45)
≥12	36 (55)
Diagnosis (*n*, %)	
Acute lymphoblastic leukaemia	29 (50)
Wilms' tumour	6 (10.1)
Acute myelogenous leukaemia	3 (5.2)
Non-Hodgkin's lymphoma	3 (5.2)
Other diagnosis	25 (29.5)
Asked to join treatment trial (*n*, %)	55 (83.33)
Treatment programme (*n*, % parents)	
Randomised	49 (74)
Non-randomised	17 (26)

GCSE/CSE, General Certificate of Secondary Education/Certificated of Secondary Education.

### Previous knowledge and information preferences

Parents reported being happy with their involvement in their child's treatment decision (mean 4.48, possible range: 1–5). Nearly 70% of the parents were content with the information provided to them at the time of diagnosis. Nine parents (13.63%) wanted more information about the medications, three (4.55%) wanted more details about the trial, one parent wanted more disease-specific information and one mother wanted a better explanation of her child's prognosis. The median time available for decision making as reported by parents was 2.33 days.

Two-thirds of the parents did not know about cancer treatments or cancer research before their child's diagnosis (*n*= 41). For those who did know, sources of previous information were: media (*n*= 10), families of other children with cancer (*n*= 4), relative/friend (*n*= 3), a charity (*n*= 3), other medical staff (*n*= 2); and one response each for school, leaflet and newsletter. Individual question items from the Decisional Conflict Scale and Satisfaction with Involvement are presented in Table 2.

**Table 2:** Decisional conflict scale and satisfaction with involvement scores

Variables	*n*	Mean	SD
Q1. The decision was hard to make	66	3.39	1.35
Q2. I was unsure what to do in this decision	66	3.05	1.18
Q3. It was clear which choice was best	66	1.89	1.00
Q4. I felt pressure from others	66	2.02	0.96
Q5. I had the right amount of support from others	55	1.69	0.71
Q6. Parents should be involved in the treatment decision	66	1.51	0.75
Q7. I feel I made an informed choice	66	1.71	0.74
Q8. I am satisfied with my decision	66	1.44	0.56
Total decision uncertainty (Q1 + Q2 + Q3)	66	8.31	2.37
Total conflict (sum of Q1 thru Q8)	66	16.68	3.44
Satisfaction with involvement	66	4.47	0.83

### Demographics and participation in decisions

For analysis of the association between parents' demographics and their participation, the data from all 66 parents were used. Parents with a higher educational attainment tended to report that they had a shorter time frame available to make their decisions (*r*= 0.249, *P* < 0.05). Younger parents both received and desired more family involvement in treatment decisions (*r*=−0.299, *P* < 0.05 for both) while older parents both received and wanted more input from other medical staff members (*r*= 0.258, *P* < 0.05 and *r*= 0.260, *P* < 0.05 respectively).

### Correlation analyses

Spearman's rho correlations were conducted for the Decisional Conflict Scale, the Responsibility for Treatment Choice Questionnaire, parental demographics and satisfaction with involvement. In partial support of the first hypothesis of the study, parental satisfaction with involvement was negatively correlated with the Total Conflict Score which was derived from the sum of eight questions in the Decisional Conflict Scale (*r*=−0.425, *P* < 0.01). However, neither the parents' nor other parties' level of involvement in the treatment decision correlated with the parents' satisfaction with their involvement. There were also no significant correlations between actual or desired participation and parental satisfaction with their involvement in the decision-making process. Therefore, the first hypothesis was not fully supported.

The second hypothesis was supported in that parental satisfaction with their decision and their involvement in decision making correlated with the belief that they had made an informed choice (*r*= 0.749, *P* < 0.001 and *r*=−0.386, *P* < 0.01 respectively). Furthermore, parents who believed the decision was easier to make reported that they had made an informed choice whereas those who were unsure which treatment to choose had higher scores on the Decisional Conflict Scale thus reported more difficulty with their decision making (*r*=−0.308, *P* < 0.05 and *r*= 0.532, *P* < 0.001 respectively).

### Patterns of decision participation

The mean reported treatment decision participation percentages were 62.47% for doctors, 29.42% for parents and 8.11% for children, other family members and healthcare staff (e.g. nurses and primary care physicians) combined. On average, the desired participation percentages were 59.30% for doctors, 32.60% for parents and 8.16% for the others combined. The participation of the doctor in the decision was negatively related to the input of the parents (*r*=−0.837, *P* < 0.001), the paediatric patients (r =−0.383, *P* < 0.01) and other medical staff (e.g. nurses; *r*=−0.350, *P* < 0.01). There was also a trend for a negative association between the doctor's participation and that of other family members (*r*=−0.252, *P*= 0.057).

### Decision participation preferences

Paired sample *t*-tests indicated that individually, parents preferred to participate more than they were able to, and that they desired less doctor participation (*t*(65) =−2.479, *P* < 0.05 and *t*(65) = 2.396, *P* < 0.05 respectively) even though there was only about 3% difference between the actual and preferred parental and doctoral participation percentages overall. Parents were more involved in the decision if they had more time to consider the options (*r*= 0.390, *P* < 0.01), while doctors tended to assume the majority of responsibility for decision making in those situations with minimal time available (*r*=−0.322, *P* < 0.05). Parents who rated the decision as difficult to make reported less involvement from doctors (*r*=−0.282, *P* < 0.05).

### Multivariate analyses

Multivariate analyses of variance were conducted to determine the effect of previous knowledge, trial status, and the passage of time on parents' decision-making experience. Parents who had no previous knowledge of paediatric cancer therapy or clinical trials preferred to have more involvement in the medical decisions regarding their child's treatment (*F*_2,53_= 6.590, *P* < 0.01). Conversely, parents with previous knowledge tended to desire more participation from the oncologist (*F*_2,53_= 3.446, *P* < 0.05, least significant difference = 0.077). A 2 × 2 analysis of variance indicated an interaction between parental gender and previous knowledge of paediatric cancer treatments for the reported and desired levels of doctor participation (*F*_1,65_= 5.784, *P* < 0.05 and *F*_1,65_= 5.456, *P* < 0.05 respectively). Specifically, mothers with previous knowledge reported and preferred more doctor involvement (*t*(58) =−2.339, *P* < 0.05 and *t*(58) =−3.147, *P* < 0.01).

Those who were asked to participate in a non-randomised protocol reported feeling more pressure from others (*F*_1,66_= 8.921, *P* < 0.01; child's age at diagnosis as covariate). They also included and desired their child's participation in the decision more than those who were asked to join randomised trials regardless of the child's age (*F*_1,58_= 5.546, *P* < 0.05). Fathers tended to report feeling more pressure from others and reported more uncertainty about the optimum choice than did mothers (*F*_1,66_= 3.334, *P*= 0.073 and *F*_1,66_= 4.946, *P* < 0.05 respectively with child's age at diagnosis as covariate).

A year after having made a treatment decision, parents were less sure about which treatment choice was best (*F*_1,66_= 4.889, *P* < 0.05 with child's age at diagnosis as covariate). Multiple regression analyses were conducted to determine the predictors of parental satisfaction with their decision and their involvement. Confidence that they had made an informed choice, and the degree of support from others during the decision-making process, were both important factors in overall parental satisfaction (see Table 3). These findings supported the study hypotheses.

**Table 3:** Regression analyses predicting parental satisfaction and support

Variables entered		*R*	*R*^2^	d.f.
*F* for model	*R*^2^ change			
Parental satisfaction with decision				
Informed choice		721***	0.520	1.64
69.3689	0.520			
Parental satisfaction with involvement				
Support from others		0.591***	0.350	1.64
34.4029	0.350			
Parental belief of informed choice				
Satisfaction with decision		0.721***	0.520	1.64
69.368	0.520			
Support from others		0.765**	0.571	1.64
7.506	0.051			
Difficulty of decision		0.780*	0.608	1.64
5.848	0.037			
Parental report of support from others				
Satisfaction with involvement		0.591***	0.350	1.64
34.402	0.350			
Informed choice		0.702***	0.493	1.64
17.834	0.143			

* *P* < 0.05

** *P* < 0.01

*** *P* < 0.001.

## DISCUSSION

### Participation

Parents believe in the importance of participating in the decisions affecting the course of their child's treatment. However, in the medical setting, doctors often take a paternalistic position in parent/physician interactions, and therefore assume most of the power in decision making (Coulter 1997). Recent research suggests that staff underestimate the level of involvement and independence that parents wish to exert on the decision-making process, and caretaking during a child's illness (Shields *et al.* 2004). Thus, family and doctor roles must be negotiated in order for optimum co-operation to take place (Thorne & Robinson 1988).

In the current study, parents stated that they would have preferred more involvement in decisions about treatment than they actually experienced. Not surprisingly, the oncologist's involvement decreased when parents assumed more of the responsibility in deciding upon their child's treatment. The doctors tended to have less involvement when the paediatric patient, other family members and other medical staff where included in the decision. This difference in involvement could be due to parents adopting a more active role in medical decision making by seeking advice from other parties. As a result, the actual degree of participation for the child, other family members and other medical staff were essentially the desired degree of participation because parents had made the decision to seek their input at the very beginning of the decision-making process.

Parents who reported less doctor participation also stated that the decision was more difficult to make. They also reported more problems with choosing a treatment when their child and family were included. It is not clear whether the participation of the other parties made the decision more difficult or if the parents chose to include them because they could not decide themselves. For example, parents reported more pressure from others and greater child participation in the decision to enter a non-randomised trial than for those choices that involved randomisation to a treatment protocol. These parents may have been more likely to include their children in decisions about non-randomised treatments as a method of reassurance or because it was easier to explain the non-randomised treatment options to the young patient.

Parents were more involved when they had more time to make a decision regarding their child's treatment. Parents who found themselves with little time to make a decision may have relegated the decision to the oncologist in an effort to eliminate the stress of having to choose a treatment when they were already under the considerable strain of coping with their child's cancer diagnosis. When parents have more time to consider the treatment options as well as the opinions of others, they may perceive themselves as more capable of taking an active role in the choosing a treatment plan.

Parental age was related to who the parents opted to include in the decision-making process. Younger parents, who are arguably less experienced in serious medical situations, included other family members more often than their older counterparts. Relatives could provide these younger parents with the additional information and support they needed to make the final decision. Conversely, older parents sought out the opinions of others in the medical community. These parents may have been more confident in their decision-making abilities than younger parents and therefore depended less on their extended family's input. They also may have had more opportunities to interact with different health professionals in their lifetime, and thus have a better knowledge of the healthcare system, or even been raised with the belief that ‘doctor knows best’. This specific type of experience could be an incentive to ask a trusted family physician for advice about entering a clinical trial instead of relying solely on the opinion of the oncologist.

Parents with prior knowledge of paediatric cancer treatments, particularly the mothers, preferred more involvement from their child's oncologist. Thus, they may have sought the professional opinion to a greater degree in an effort to receive optimum care with fewer side effects. Conversely, those parents who did not have prior knowledge of what cancer therapies were available wanted to be more involved in the decisions affecting their children's treatment plan. This preference may be due to a ‘closing of ranks’ following such a serious diagnosis whereby parents rely solely on their family coping strategies instead of seeking outside support. To date, little research has been done on the levels of information patients' families have regarding their illness prior to diagnosis. Degner and Sloan (1992) found, however, that those patients who were recently diagnosed with cancer needed less involvement in cancer treatment decisions when compared with the general public. Thus, those who had attained more disease and treatment-specific information (i.e. the patients) wanted more doctor participation, much like the parents who had prior knowledge of the details of paediatric cancer treatments in this study.

### Satisfaction

Parents indicated that when there was less conflict, they were more satisfied with their choice and their involvement in the decision-making process. ‘Conflict’ included factors such as pressure from others and the clarity of choice. Parents were happier with their decision when they felt that they had made an informed choice and received the right amount of support. In relation to information, parents rated support in terms of how willing medical staff were to give information regarding treatment options.

Perceived support predicted the parents' satisfaction with their involvement. The opportunity to discuss treatment options with medical personnel may have helped alleviate some of the concerns of the parents. Parents assume responsibility for their child's health care. Exclusion from the treatment decision-making process would be in conflict with their desired caregiver role (Pinkerton *et al.* 1998). Thus, the efforts of medical staff to involve parents in choosing from different treatment options would have a favourable outcome on parental satisfaction as it is consistent with their caregiving role.

The manner in which parents were involved in decision making also impacted upon their level of satisfaction. Although their actual degree of participation did not correlate with their overall satisfaction, parents believed that they should be more involved in their child's treatment decisions when it was less evident as to which choice was best. Reviewing the options and evaluating the risks could give the parents a sense of control over the health of their child.

However, the primary evaluation of the positive and negative aspects of participating in paediatric cancer research not only affects the parents' immediate decision, but could exacerbate the stress that occurs from making that choice.

Parents found the decision-making process more difficult when they were unsure what to do, indicating that their assessment of the risks and benefits may remain unresolved even when they settle upon a choice. Furthermore, parents of children at least 1 year into the treatment protocol reported that they were still unsure which treatment choice was optimal. Their increased concerns may have been the result of witnessing the effects of the treatment on their child's condition and functioning for a longer period of time. If the parents' assessment of the benefits of the treatment became clouded by the occurrence of side effects or setbacks, they may have been less certain that they had made the best treatment choice for their child.

As parental decision making in the oncology setting has only begun to be examined in recent years, much research remains to be done in order to determine which factors influence parental satisfaction with this process. For instance, this study found that information and reassurance provided by the medical staff could be used to predict the level of parental contentment with the decision-making experience overall. Parents may have been able to minimise their decisional conflict by receiving the appropriate levels of assistance from medical staff and family members. Consequently, future research should investigate when parents want to be provided with information, and to what level of detail. Whether parents require informational or emotional support must be determined in order to improve the services provided to the families. Although there were no differences between the satisfaction rates of parents within the first 12 months of the treatment plan and those more than 1 year into the chosen protocol, there may have been differences in how the parents evaluated their overall choice based on their cancer treatment experiences.

Furthermore, the needs of parents may change as their family progresses through the protocol (Kupst 1993). The type of information or support required in the early stages following diagnosis may not be sufficient to help parents with the difficulties that arise later in the treatment process. A limitation of the cross-sectional methodology employed in this study is that it cannot specify how the identified determinants of parental satisfaction change over time. Interviews with parents throughout their child's cancer treatment and follow-up consultations may help to specify how their needs evolve over time and the subsequent effect of these upon their satisfaction. Indeed, taking time to listen to the concerns of families would be a method of providing support that could improve their overall satisfaction levels (Goore *et al.* 2001), and new mechanisms for achieving this level of care are currently being trialled, such as videotelephony to improve clinical and psychosocial support (Bensink *et al.* 2007). These basic supportive measures may help lower parental anxiety regarding their choice and improve the families' relationship with the medical staff throughout the course of their children's care.

### Limitations

The main limitations of the study are that reported levels of parental involvement do not necessarily reflect the actual participation patterns that took place during the decision-making process. However, the difference between what parents desire and what they believe they receive will impact on satisfaction levels. These in turn can change, but the study was not able to explore how satisfaction, nor how determinants of parental satisfaction, change over time.

Further research should be carried out into whether the participation of an increased number of other parties makes the decision making more difficult or if the parents chose to include them because they cannot make such decisions by themselves. If future research examines which factors influence parental satisfaction, the current findings suggest that a worthwhile area to investigate is when parents want to be provided with information and at what level of detail. Last, this may in turn inform, or be informed by, research examining the impact of differing levels of information about the illness that patient families have prior to their diagnosis.

### Implications for practice

There are several important clinical implications of this study. First, medical staff should be aware that being asked to take part in a non-randomised trial results in parents reporting more pressure from others and potentially more stress during the decision-making process. Furthermore, parents were more involved when they had more time to make a decision regarding their child's treatment so, where clinically possible, sufficient time should be made for parents to consider the options fully. Last, the willingness of medical staff to provide information was a key factor in perceived support, which in turn was associated with parents' satisfaction with their involvement in the decision-making process. Thus medical and nursing professionals need to ensure that parents know they are readily available to answer questions and prepared to provide additional information as required.

## Conclusion

A diagnosis of childhood cancer and its subsequent treatment demands provide complex challenges for families as they attempt to adjust to a ‘new norm’ of family functioning. Reassurance from medical staff that the parents' questions are important throughout the decision-making process is crucial to establishing a good working relationship between the medical team and parents which will last the duration of the child's treatment.
